# Modified dorsal root entry zone lesioning for pain relief in cervical root avulsion injury

**DOI:** 10.3171/2020.7.FOCVID2020

**Published:** 2020-10-01

**Authors:** Keisuke Takai, Makoto Taniguchi

**Affiliations:** Department of Neurosurgery, Tokyo Metropolitan Neurological Hospital, Tokyo, Japan

**Keywords:** brachial plexus avulsion, deafferentated pain, dorsal root entry zone, posterior horn

## Abstract

Neuropathic pain in the upper extremity due to cervical root avulsion injury is refractory to medical treatments. Superficial layers in the posterior horn of spinal gray matter, including the substantia gelatinosa, are the main target of dorsal root entry zone (DREZ) lesioning, which has been the most effective surgical treatment for the relief of intractable pain; however, residual pain and a decrease in pain relief during the long-term follow-up period have been reported. Based on pain topography in the most recent basic studies, the conventional DREZ lesioning procedure was modified to improve clinical outcomes.

The video can be found here: https://youtu.be/PyaAGmAE7Og

## Transcript

We herein demonstrate our modified dorsal root entry zone lesioning procedure for pain relief in a case of cervical root avulsion injury.

**0:32 The Anatomy and Pathways of the Posterior Horn.** Illustrations (Supplemental Fig. 1) show the anatomy (left) and pathways (right) of the posterior horn of a healthy spinal cord. The posterior horn of spinal gray matter comprises six regions, the laminae of Rexed I–VI (left). Neurons in the deep layers are called wide-dynamic-range neurons (right), which play a role in pain conduction.^1^

**1:06 Original DREZ Procedures of Nashold and Sindou.** Illustrations (Supplemental Fig. 2) show the original procedures of Nashold (left) and Sindou (right).^2,3^ Both procedures aim at lesioning the superficial layers. The limitations of these procedures are residual pain and pain recurrence in long-term follow-up periods.^3,4^

**1:31 Modified DREZ Lesioning.** In contrast, our modified procedure lesions deeper layers than Nashold’s and Sindou’s procedures based on recently reported pain topography.^5^ We started performing this technique for cervical root avulsion injury in 2011. Over the past decade, 30 cases have been treated at Tokyo Metropolitan Neurological Hospital, achieving total and persistent pain relief.

**2:07 Clinical Presentation.** A 65-year-old man presented with left arm pain at the C6–8 levels. He had a motorbike accident 25 years before his admission to our hospital. Pain gradually worsened after the accident; however, no medical treatment was effective.

**2:31 Positioning and Approach.** The patient was positioned prone, and transcranial motor evoked potentials in the ipsilateral lower extremity were monitored intraoperatively to detect damage to the corticospinal tract in the lateral funiculus. Cervical multilevel hemilaminotomy was performed.

**2:58 Localization of DREZ.** Anatomical landmarks to localize the DREZ are the residual spinal roots and posterior spinal arteries (Supplemental Fig. 3). In this case, the upper and lower limits of lesioning were at the C5 and T1 posterior nerve roots in the DREZ.

**3:22 Dissection of DREZ.** After opening the dura mater, the T1 and C5 posterior roots were detected. Subtle defects due to avulsion injury were observed. The pia mater of the DREZ was incised between the C5 and T1 roots. The spinal cord was divided into the posterior and lateral funiculus using a microsurgical probe.

The anatomical landmarks to define the dissection plane between the posterior and lateral funiculus were the posterior sulcal veins in the DREZ. Care is needed to avoid damaging the fiber bundles of the spinal cord. Spinal arteries and veins were left intact as much as possible.

**4:26 Modified DREZ Lesioning Technique.** When the posterior and lateral funiculi were completely divided, the depth of lesioning reached deep layers. The posterior horn was lesioned using microsurgical forceps with a blunt dissection technique at a depth of 4–5 mm from the surface of the DREZ. The depth of lesioning was confirmed using a small paper ruler.

**4:56 Closure.** After dural closure, the laminae were fixed with mini-plates.

**5:00 Postoperative MRI and Outcome.** Postoperative MRI shows the site of DREZ lesioning. Complete pain relief was achieved immediately after surgery without motor or sensory deficits. No recurrence was reported at the 5-year follow-up.

**5:20 References**

## Author Contributions

Primary surgeon: Taniguchi. Assistant surgeon: Takai. Editing and drafting the video and abstract: Takai. Reviewed submitted version of the work: Takai. Approved the final version of the work on behalf of both authors: Takai. Supervision: Takai.

## Supplemental Information

### Online-Only Content

Supplemental material is available online.

*Supplemental Figs. 1–3*. https://thejns.org/doi/suppl/10.3171/2020.7.FOCVID2020.

## Supplemental Information

Supplemental Figures
