# Supplementary material for: Modified dorsal root entry zone lesioning for pain relief in cervical root avulsion injury
Source: Neurosurg Focus Video. 2020 Oct 1;3(2):V11. doi: 10.3171/2020.7.FOCVID2020 (PMC9542433; doi:10.3171/2020.7.FOCVID2020)
Supplement: Supplemental Figures [file SupplementalFigs1-3_FOCVID20-20.pdf]

NEUROSURGICAL FOCUS VIDEO

ONLINE ONLY

Supplemental material

Modified dorsal root entry zone lesioning for pain relief in cervical root avulsion injury

Takai et al.

https://thejns.org/doi/abs/10.3171/2020.7.FOCVID2020

DISCLAIMER The *Journal of Neurosurgery* acknowledges that the following section is published verbatim as submitted by the authors and did not go through either the *Journal's* peer-review or editing process.

© 2020 The authors, CC BY 4.0 (http://creativecommons.org/licenses/by/4.0/)

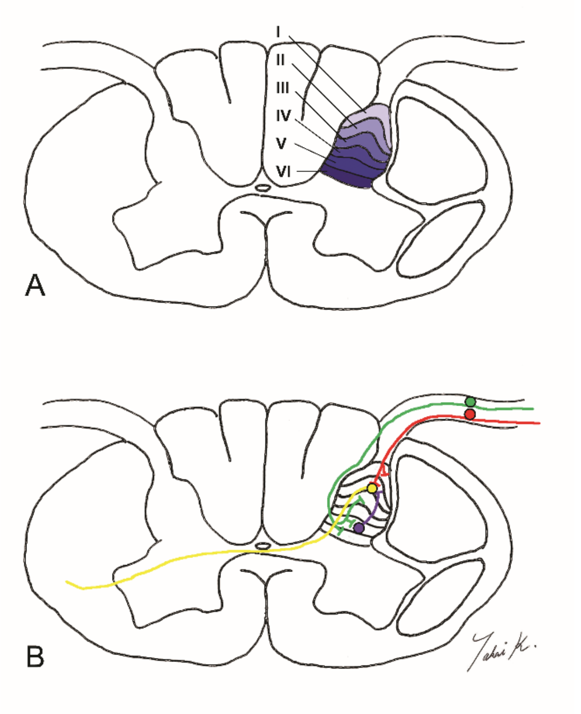

FIG. 1. Illustrations showing the anatomy of the posterior horn (**A**) and primary and secondary afferent pathways in the posterior horn of healthy spinal gray matter (**B**). The posterior horn of spinal gray matter comprises 6 layers, the laminae of Rexed I–VI (A). The thick C-fibers of nociceptive neurons (red) project to cells in the laminae of Rexed I and II, and connect with the secondary afferent neurons of the contralateral spinothalamic tract (yellow). The narrow A-fibers of the nonnociceptive neurons (green) project to cells in the laminae of Rexed III–VI. Secondary neurons in the deeper lamina of Rexed V (purple) are called wide dynamic range neurons because they receive synaptic inputs from A-fibers, connect with the contralateral spinothalamic tract, and are associated with pain conduction. Copyright Keisuke Takai. Published with permission.

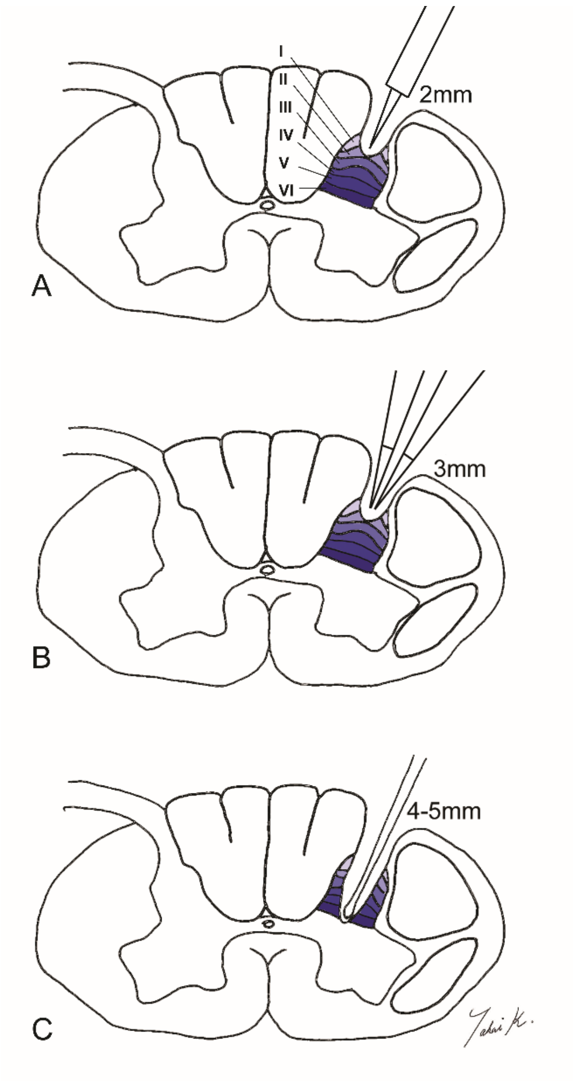

FIG. 2. Illustrations showing the depth of DREZ lesioning of the procedures of Nashold (**A**) and Sindou (**B**), as well as our procedure (C). Nashold's and Sindou's procedures aim at lesioning cells in the substantia gelatinosa in the laminae of Rexed I and II. In contrast, our procedure aims at lesioning the deeper layers of the posterior horn of spinal gray matter, including the lamina of Rexed V. In the healthy spinal cord, the depth of the lamina of Rexed II is 2–3 mm (A and B), whereas that of the lamina of Rexed VI is 4–5 mm (C) from the surface of the DREZ. Copyright Keisuke Takai. Published with permission.

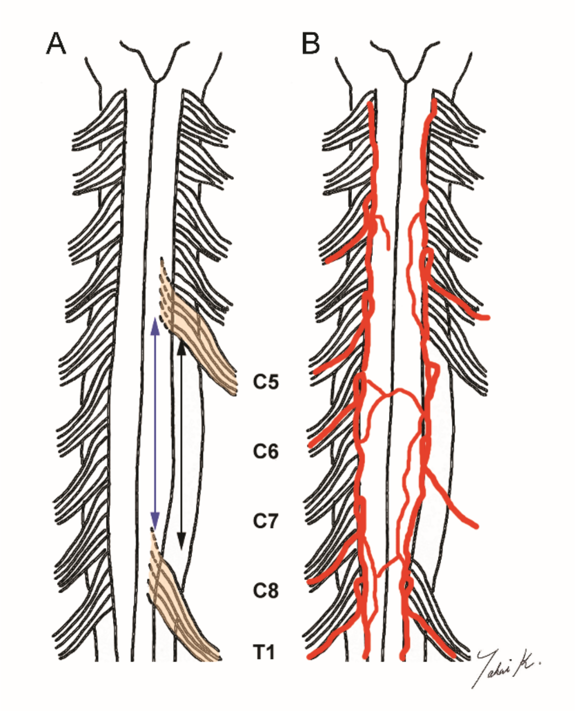

FIG. 3. Illustrations showing the sagittal range of DREZ lesioning (**A**) and relevant vascular anatomy (**B**) in a case of root avulsion injury at the level of C6–8. The pia mater of the intermediolateral sulcus is incised between the healthy spinal roots (black double-headed arrow). The proximal portions of posterior nerve roots are located within the DREZ. The upper and lower limits of lesioning are at the proximal portion of intact C-5 and T-1 posterior nerve roots in the DREZ (blue double-headed arrow). A radiculopial artery courses along the spinal root, turns a hairpin curve, and joins the posterior spinal artery (B). The radiculopial artery is observed in some cases of root avulsion injury. When the intermediolateral sulcus is divided, the radiculopial and posterior spinal arteries are preserved intact as much as possible. Copyright Keisuke Takai. Published with permission.
